# Species-wide Metabolic Interaction Network for Understanding Natural Lignocellulose Digestion in Termite Gut Microbiota

**DOI:** 10.1038/s41598-019-52843-w

**Published:** 2019-11-08

**Authors:** Pritam Kundu, Bharat Manna, Subham Majumder, Amit Ghosh

**Affiliations:** 1 School of Energy Science and Engineering, Indian Institute of Technology Kharagpur, Kharagpur, West Bengal 721302 India; 2 P.K. Sinha Centre for Bioenergy and Renewables, Indian Institute of Technology Kharagpur, Kharagpur, West Bengal 721302 India

**Keywords:** Computational biology and bioinformatics, Systems biology

## Abstract

The structural complexity of lignocellulosic biomass hinders the extraction of cellulose, and it has remained a challenge for decades in the biofuel production process. However, wood-feeding organisms like termite have developed an efficient natural lignocellulolytic system with the help of specialized gut microbial symbionts. Despite having an enormous amount of high-throughput metagenomic data, specific contributions of each individual microbe to achieve this lignocellulolytic functionality remains unclear. The metabolic cross-communication and interdependence that drives the community structure inside the gut microbiota are yet to be explored. We have contrived a species-wide metabolic interaction network of the termite gut-microbiome to have a system-level understanding of metabolic communication. Metagenomic data of *Nasutitermes corniger* have been analyzed to identify microbial communities in different gut segments. A comprehensive metabolic cross-feeding network of 205 microbes and 265 metabolites was developed using published experimental data. Reconstruction of inter-species influence network elucidated the role of 37 influential microbes to maintain a stable and functional microbiota. Furthermore, in order to understand the natural lignocellulose digestion inside *N*. *corniger* gut, the metabolic functionality of each influencer was assessed, which further elucidated 15 crucial hemicellulolytic microbes and their corresponding enzyme machinery.

## Introduction

A substantial increase in global energy demand along with the climatic deterioration, has motivated the advancement of alternative and sustainable energy source. Consequently, biofuels, the thriving candidates to replace petroleum-derived fuels, have attracted a great interest worldwide^1^. Lignocellulosic bio-polymer with an enormous amount of carbohydrate contents can provide a massive source of feedstock for the production of biofuels^2^. However, the structural and compositional complexities of the cellulose, hemicellulose, and lignin in the biomass restrict its deconstruction into fermentable sugar^3^. Pretreatment removes the physicochemical obstacles in raw biomass, leaving cellulose and hemicellulose vulnerable to enzymatic depolymerization^4^. Although the enzymatic hydrolysis of these plant-derived polymers is an effective method for the extraction of fermentable sugars, it has a high economical constraint^5^.

*Nasutitermes corniger*, a wood-feeding higher termite with natural lignocellulose digestion system, can effectively remove 74–99% of cellulose and 65–87% of hemicellulose from woody biomass, leaving the lignin-rich residues as faeces^6^. Diverse microbial communities residing in distinct gut compartments of *N*. *corniger* are responsible for developing this remarkable system of lignocellulose deconstruction and subsequent fermentation^7^. Each microbial entity is metabolically interacting with diverse community members through the exchange of small metabolites^8^. Specific interactions of these naturally viable consortia cause diverse metabolic phenomena like commensalism, amensalism, co-operation, competition, and predation^9^. Metabolically communicating microbial symbionts and their enzyme machinery are necessary to accomplish the complex functionality of lignocellulose degradation inside the termite gut.

Earlier studies on metagenomics and functional analysis of termite gut microbiota provided crucial insights into predominating microbial genera, for lignocellulose digestion^10–12^. A broad range of glycoside hydrolases (GHs) for efficient depolymerization of cellulose have also been identified^13,14^. However, even though the advanced techniques of metagenomics and metatranscriptomics provide vast genomic and taxonomic information, the pattern of species-wide metabolic interaction remains unclear. Numerous strategies of microbial interactions have been developed based on the statistical correlations of taxonomic abundances or by mapping the entire metabolic pathways through direct metagenome annotation^15–17^. However, the correlation-based inference networks and the assessment of community-wide metabolic capabilities, both failed to describe the precise mechanisms of inter-species interactions. Moreover, how the combinational influences of different microbes in a multi-species cross-feeding arrangement develop a community level function of lignocellulose digestion is still obscure.

This study focuses on understanding the microbial metabolic cross-communication and the mechanism of lignocellulose digestion in *N*. *corniger* gut by constructing a species-wide metabolic interaction network. The shotgun metagenomic data was used to identify microbial species in distinct gut compartments of *N*. *corniger*. Literature-curated information of the microbial metabolic activities was taken as elementary components to frame the network architecture. Moreover, the inter-species influence network demonstrates the substantial effect of various metabolic functionality in a complex microbial niche. The metabolic profiles of several microbial influencers have been characterized to decipher the role of key microbial players in a stable and functional microbiota. Moreover, to understand the natural bioreactor system inside *N*. *corniger* gut, a set of enzyme cocktail has been exemplified with corresponding lignocellulose degraders.

## Results

### Construction of species-wide metabolic interaction network of N. corniger’s gut microbiota

**Figure 1:**
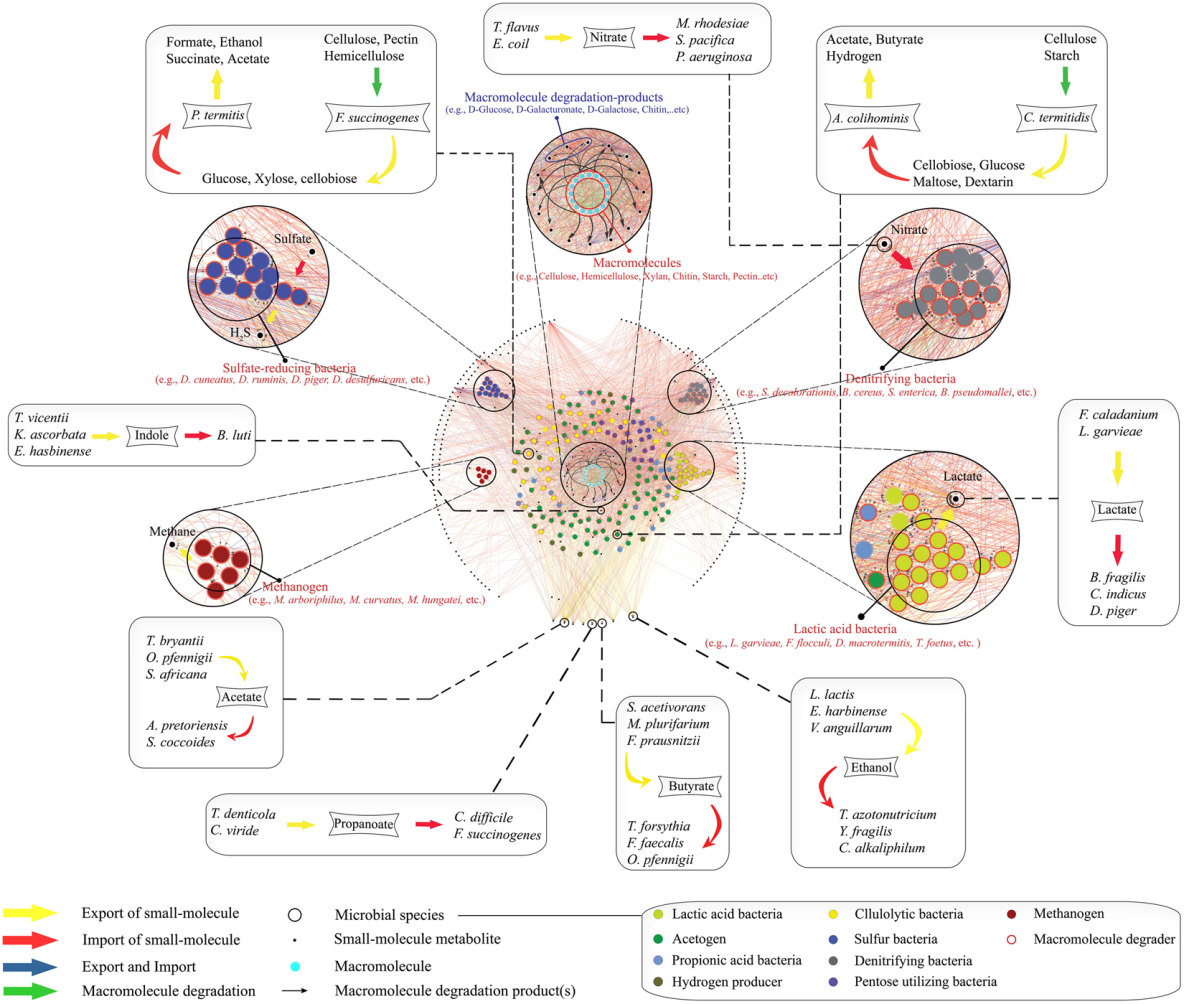
Species-wide metabolic interaction network in *N*. *corniger* gut microbiota. The species-wide metabolic interaction network consists of three distinct classes of nodes to denote microbial entities (eight large colored nodes), small metabolites (small black nodes), and macromolecules (medium cyan nodes). Small molecules are distributed at the periphery of the network while the macromolecules are represented at the center. Microbes with similar metabolic activities were shown in eight distinct groups *i*.*e*., lactic acid bacteria (light green), acetogen (dark green), propionic acid bacteria (sky blue), hydrogen producer (olive green), cellulolytic bacteria (yellow), sulfur bacteria (blue), denitrifying bacteria (grey), and pentose utilizer (dark purple). Some relevant groups are exemplified to get a better resolution of their metabolic characteristics. The nodes of macromolecule degraders are encircled in red to distinguish them from other microbial entities. Microbes are connected to the metabolites with corresponding metabolic activities, *i*.*e*., import (red edges), export (yellow edges), both import-export (blue edges), and macromolecule degradation (green edges). Magnified circle of macromolecule degradation shows the connections between macromolecule and their degradation products. Several metabolic activities of distinct microbial entities can be seen in rectangular boxes, *e*.*g*., *F*. *succinogenes* depolymerize cellulose, pectin, and hemicellulose to supply small metabolites such as xylose, glucose, cellobiose, and mannose, which are consumed by *R*. *bormii* to produce fermentative byproducts like acetate, ethanol, succinate, and formate in the microenvironment. The network was developed using Cytoscape v3.6.1^59^.

**Figure 2:**
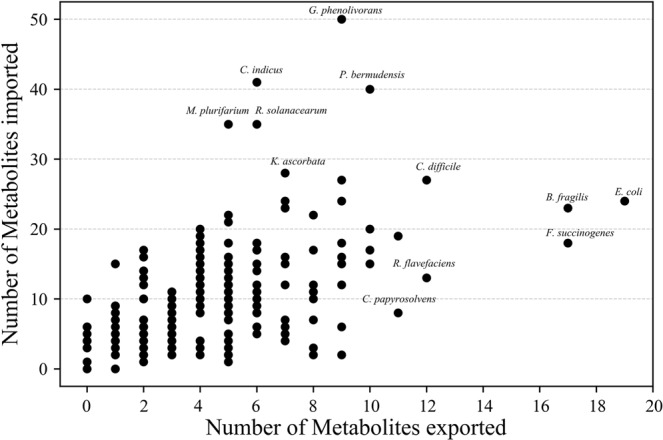
Metabolic transportation profile of the individual microbial entity. Metabolites import and export frequency of each microbial entity has been represented using black scattered dots. The vertical and horizontal axis denotes the number of imported and exported metabolites, respectively. It is observed that the number of small metabolites transmission varies significantly among different microbial species. Moreover, the frequency of metabolite import is found to be higher than the export inside the *N*. *corniger* gut microbiota. The statistical overview of the network has been provided in the Supplementary Information (Table S5).

Shotgun metagenome sequencing data of *N*. *corniger’s* gut were analyzed to get the species-level information of microbial composition in the distinct gut compartments. An extensive literature survey was carried out with identified microbes to obtain the information of metabolite import and/or export activities (Supplementary Data). Collectively, 2988 annotated metabolic events (import and/or export) of 205 identified species and 265 metabolites were included for the construction of species-wide metabolic interaction network (Fig. 1). Evaluation of the structural properties of the network was also carried out to quantify the metabolic transportation activities inside the termite gut microbiota (Fig. S1). The average values of metabolite consumption and production by 205 microbes were calculated to be 10.5 and 4.6, respectively (median - 9 and 4, respectively). The quantitative measure of metabolite transport indicated that the capability of importing or exporting different metabolites varied widely among microbial entities (Fig. 2). Promising microbes such as *Granulicoccus phenolivorans* (imports 50 and exports 9 metabolites), *Pelagibaca bermudensis* (imports 40 and exports 10 metabolites), and *Fibrobacter succinogenes* (imports 18 and exports 17 metabolites), attained a high metabolic import and/or export activity. The correlation coefficient (R^2^ = 0.16) between microbial abundance and their import-export profile indicate a random distribution of microbial species inside the termite gut (Fig. S2, Table S1). Glucose, maltose, xylose, and cellobiose were found to be the most influential substrates, incorporated by 69%, 46%, 43%, and 42% microbial species, respectively. Important metabolic byproducts like acetate, lactate, ethanol, H_2_, and CO_2_ were commonly produced and exported to the gut microenvironment. Notably, acetate was the most frequently generated metabolic byproduct, produced by 53% of the microbial population. In addition, metabolites that are rarely produced by the microbial populations, like putrescine, methanethiol, 4-aminobutyrate (GABA), trimethylamine, urea, and lithocholic acid were also encountered inside the gut microbiota. For example, methanethiol production by the *Sporobacter termitidis and Parasporobacterium paucivorans* provided insight about the sulfur metabolism and the degradation of methoxylated aromatic compounds inside the gut microbial niche^18–20^. Similarly, *Pseudomonas aeruginosa and Escherichia coli* produced 4-aminobutyrate which is a substantial element of the free amino acid pool in most prokaryotes and eukaryotes^21^.

Collective metabolic activities of these diverse microbial groups maintain a functional gut microbiota^22^. In order to understand the organization of these metabolically interacting groups, the correlation of microbial co-occurrence score and their metabolic similarity indices has been investigated by calculating Spearman’s correlation coefficient^23,24^. The co-occurrence score and metabolic similarity index showed a positive correlation (rho = 0.235, *p* value = 1.115 × 10^−7^) between lignocellulose degrading bacteria and ethanol producing bacteria (Supplementary Information, Page 2). This phenomena of metabolic interdependency gives rise to the positive co-occurrence between these microbial groups. For example, cellulose and hemicellulose degraders (*Candidatus Solibacter usitatus* and *Clostridium termitidis)* supplied breakdown products, such as glucose, fructose, arabinose, and xylose, in the microenvironment that have been utilized by several metabolically dependent microbes like *Treponema azotonutricium* and *Candida arabinofermentans*. Metabolic interdependency was also present in the form of inter-species cross-feeding; for instance, succinate produced by *Fibrobacter succinogenes* and *Bacteroides fragiles* was utilized by *Bacteroides coprosuis and Pseudomonas aeruginosa*.

Conversely, microbial groups with similar metabolic profiles sometime compete with each other to get better access to a common metabolite^25,26^. However, in this study, microbial metabolic similarity index and the co-occurrence score of two metabolically similar groups, *i*.*e*., lactic acid bacteria and propionic acid bacteria indicated a considerable inter-dependence (rho = 0.305, *p* value 3.29 × 10^−12^). This positive correlation was possibly guided by habitat filtering^27–29^, wherein the gut physicochemical environment of the host (*N*. *corniger*) played a crucial role to retain the competing microbes in a stable community. In order to maintain this stable and functional microbiota, each microbial community must have influenced the growth and abundance of other communities through diverse metabolic phenomena^30,31^. Previous studies in human gut microbiota also provided similar evidences of various metabolic activity and microbial co-occurrence pattern^15,32^.

Construction and analysis of the species-wide metabolic interaction network suggested a metabolically driven competitive and co-operative relationship among different microbial entities inside *N*. *corniger* gut microbiota. In order to assess the metabolic impact of the dominating microbial species, an inter-species influence network has been reconstructed utilizing the basic prototype of the species-wide metabolic interaction network.

### Reconstruction of inter-species influence network

**Figure 3:**
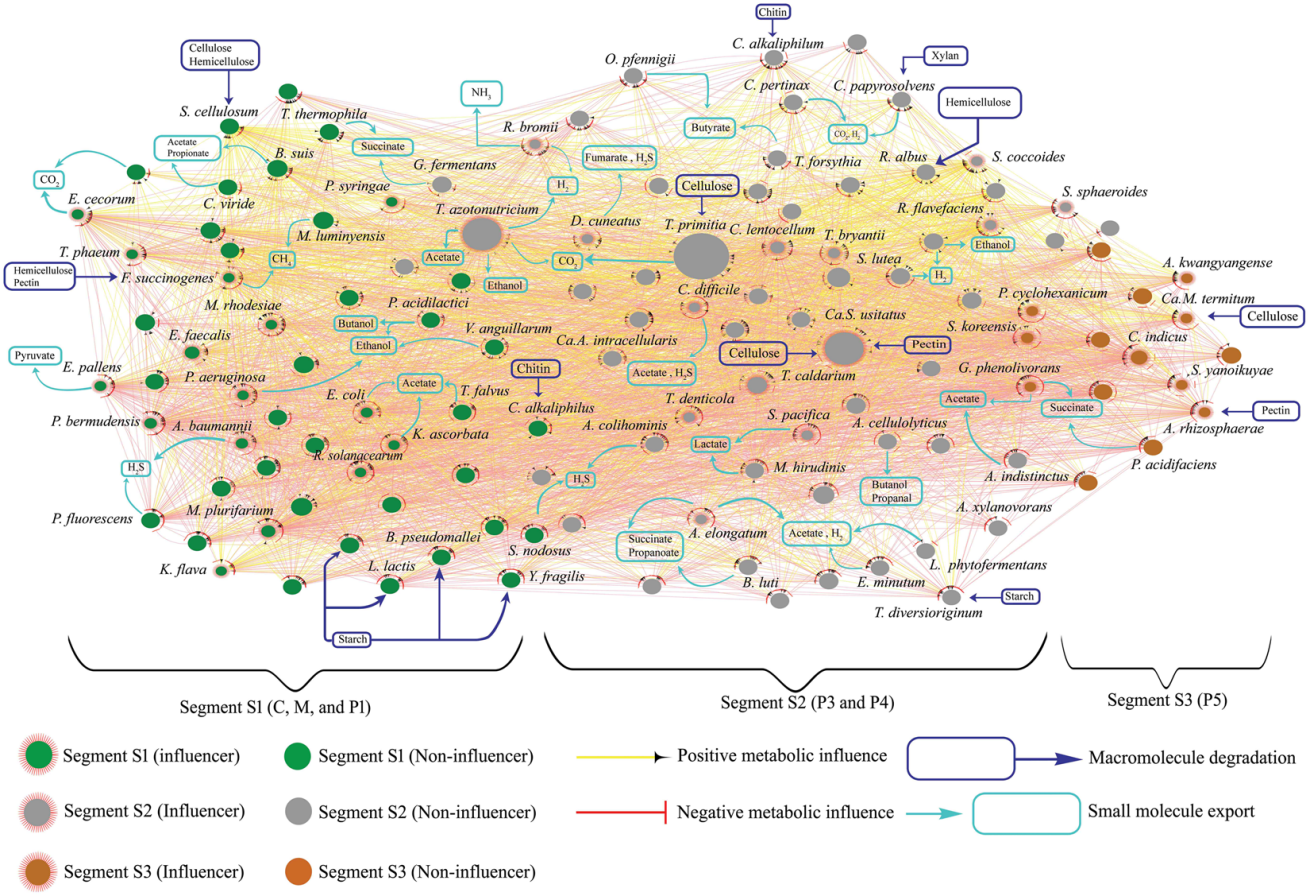
Inter-species influence network of 125 most abundant microbial entities in *N*. *corniger* gut microbiota. The network represents an inter-species metabolic influence map of abundant microbial entities in S1 (green nodes), S2 (grey nodes), and S3 (orange nodes) gut segments of *N*. *corniger*. The modes of each pairwise interaction have been characterized as positive (yellow edges) or negative (red edges) metabolic influence. Nodes with extended red background signify as network influencers that employ considerable metabolic influence over a large number of individual entities. Moreover, diverse metabolic events of small molecule export (cyan box) and macromolecule degradations (blue box) are also annotated to describe the metabolic flow in the gut microbial machinery. For instance, *M*. *rhodesiae* and *F*. *succinogenes* employ positive metabolic influence on the methanotrophic bacteria *M*. *populi* by providing methane in their microenvironment.

*N*. *corniger* has evolved with a compartmentalized gut environment harboring specialized microbial communities for effective hydrolysis of plant polymers^6,33^. Although the overall mechanism of biomass breakdown is still not clear, several hemicellulolytic microbial phyla such as *Spirochaetes*, *Bacteroidetes*, *Firmicutes*, *and Fibrobacteres*, have been identified in different gut compartments^34,35^. An inter-species influence network of *N*. *corniger’*s gut microbiota will provide useful insight into this natural biomass conversion process. More precisely, the network will unveil the capacity of each microbial species to enhance or restrain the growth of other species through diverse metabolic activities^31,32,36^. Intestinal tract of *N*. *corniger* is comprising of six major gut compartments, *i*.*e*., crop (C), midgut (M), and four major hindgut segments - P1, P3, P4, and P5^37^. However, for the reconstruction of the inter-species influence network, we have reclassified these gut segments by investigating the correlation pattern of microbial abundance variation. A positive correlation (H_stat_ = 4.268, *p* value = 0.1183, significance level 0.05) in microbial abundance distribution was observed inside the first three compartments, *i*.*e*., C, M, and P1. Similarly, the P3 and P4 segments have shown a considerable resemblance in the microbial abundance distribution pattern (H_stat_ = 1.748, *p* value = 0.1748, significance level 0.05). P5 segment was considered as a separate segments due to its low similarity with adjacent compartment (Supplementary Information, Page 4). Based on this statistical correlation, three segments, *i*.*e*., S1 (C, M, and P1), S2 (P3 and P4), and S3 (P5), were considered for the reconstruction of influence network, where the most abundant species were shown as representative microorganisms. The quantitative values (Methods, Eq. 3) of metabolic influences for each species pair have been evaluated in the form of a species-wide influence matrix (Supplementary Data, Sheet name: I_pq_ Matrix). Among all possible pair-wise influence scores (*I*_*pq*_), some highly influential interactions of 125 microbes have been represented in the influence network with 49, 61, and 15 abundant microbial species in S1, S2, and S3 segments, respectively (Fig. 3). The metabolic contributions of each microbial species have been precisely assessed to characterize the mode of their metabolic influences. Macromolecule degraders like *Ca*. *S*. *usitatus* produced a wide range of degradation products for the community feed, exerting a net positive influence over several species like *F*. *succinogenes*, *C*. *papyrosolvens*, and *R*. *albus*. The positive influences were also identified in the form of cross-feeding reactions where the metabolic byproducts of one species provided nutrients for other microorganisms. While investigating the metabolic fate of each pair-wise interaction in the microbial community, we have encountered several exchange reactions responsible for these distinct metabolic effects. For instance, *Treponema denticola* employed a net positive metabolic impact of +0.62 and +0.47 on *R*. *bromii* and *B*. *fragilis*, respectively. During the assessment of the metabolic exchange events, it was observed that *T*. *denticola* provided metabolic byproducts like lactate and valerate to *R*. *bormii* and *B*. *fragilis*, leading to positive metabolic impacts. Alternatively, negative metabolic impacts were also identified as a crucial part of the microbial influence network. For instance, *P*. *superfundia* and *P*. *cyclohexanicum* in the S3 segment employed a net negative metabolic influence (−0.19 and −0.097) on each other. Investigation of the metabolic compounds shared between *P*. *superfundia* and *P*. *cyclohexanicum* revealed that both of these microbial entities thriving on D-xylose, D-glucose, D-mannose, sucrose, and maltose. Hence, they compete with each other to get better access to these common substrates in the gut microenvironment, leading to the negative metabolic impact.

Network robustness calculation was performed by randomly reducing 25% of original data (microbial species and/or metabolic activity) followed by the reconstruction of modified inter-species influence network I and II (Figs S3 and S4). Analysis of the modified networks revealed that ~75% of influencer, including some potential members such as *Ca*. *S*. *usitatus* (*C*_*b*_ = 0.0144), *T*. *azotonutricium* (*C*_*b*_ = 0.016), and *P*. *bermudensis* (*C*_*b*_ = 0.08), have retained their influencing characteristics similar to the original influence network (Supplementary Information, Page 6). Collectively, the inter-species influence network represented numerous metabolic events in the form of macromolecule degradation and small molecule exchange to illustrate the effects of chemical cross-communication inside the gut microbial community.

### Network analysis and influencer identification

**Figure 4:**
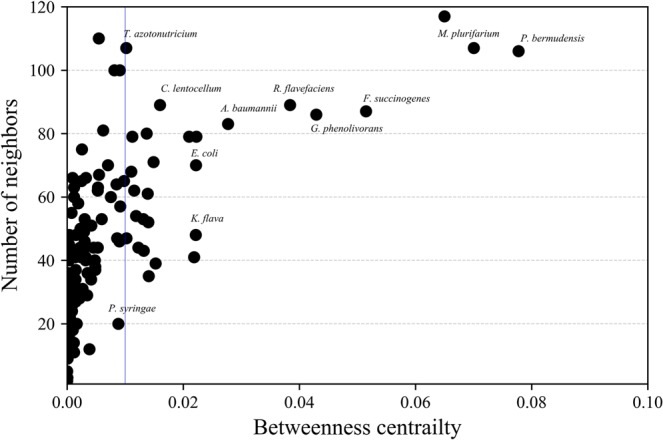
Identification of network influencer. The number of directly connected neighbors of each microbial entity in inter-species influence network were plotted as a function of betweenness centrality (*C*_*b*_) parameter. The potential network influencers were identified based on *C*_*b*_ value cutoff (>0.0074) shown on the right-hand side of the blue line. These key microbial influencers employ significant metabolic influence over a wide range of species to maintain a stable and functional microbial population inside the *N*. *corniger* gut.

Inter-species influence network suggests that the cumulative effects of positive and negative metabolic influences among microbes are essential to maintain a stable microbial consortium. Each microbe residing inside a complex microbial niche can affect the growth and viability of numerous individual species in both direct and indirect fashion. Out-degree distribution of each node was evaluated to identify the hub nodes in the directed network (Fig. S5). For instance, *Treponema primitia* and *Celeribacter indicus* have an out-degree distribution of 110 and 114 respectively, indicating a higher direct influence on the microbial communities. However, inside a composite microbial population, the out-degree distribution does not always provide significant information about the community-wide influence of an individual microbial entity. Therefore, betweenness centrality (*C*_*b*_) of each microbe was measured to identify the key players with robust metabolic control (Fig. 4). Based on the average *C*_*b*_ value, a cutoff was defined (*C*_*b*_ > 0.0074) for selecting the network influencers (Supplementary Data, Sheet name: Network analysis). Among all the microbial species represented in the influence network, 29.6% were found to be the most influential.

Detailed investigation of network influencers has been carried out to characterize their mode of influence on the community members. Assessment of the metabolic profile revealed that about 57% of the influencers were exclusively involved in macromolecule degradation, whereas about 22% microbes solely utilized sugars to produce fermentation products such as ethanol, butanol, H_2_, CO_2_, and acetate. More interestingly, 11% of the influential microbes were capable of facilitating both tasks of macromolecule degradation and fermentation. *F*. *succinogenes* (*C*_*b*_ = 0.05 and out-degree = 71) and *Ca*. *S*. *usitatus* (*C*_*b*_ = 0.008 and out-degree = 99) were found to be the most influential macromolecule degraders that exerted a substantial positive influence over 52.8% and 36.8% of total microbial species, respectively. On the other hand, *T*. *azotonutricium* (*C*_*b*_ = 0.010 and out-degree = 107), the second most abundant species in influence network, employed a negative metabolic influence over 83.2% of total microbes in the network. *T*. *azotonutricium* utilized a wide range of carbon sources such as D-glucose, D-fructose, D-ribose, D-xylose, maltose, and cellobiose, thus creating a competitive metabolic environment for other species to access these similar metabolites. This metabolic competitiveness can be the major reason behind the negative metabolic influences of *T*. *azotonutricium* over a large number of microbes. The continuous flow of metabolic byproducts in the microbial ecosystem is the basis of diverse metabolic influences. Systemic coordination of both positive and negative influences is essential to maintain a naturally viable microbial consortium.

### Distributions of common metabolic byproducts in termite gut microbial machinery

**Figure 5:**
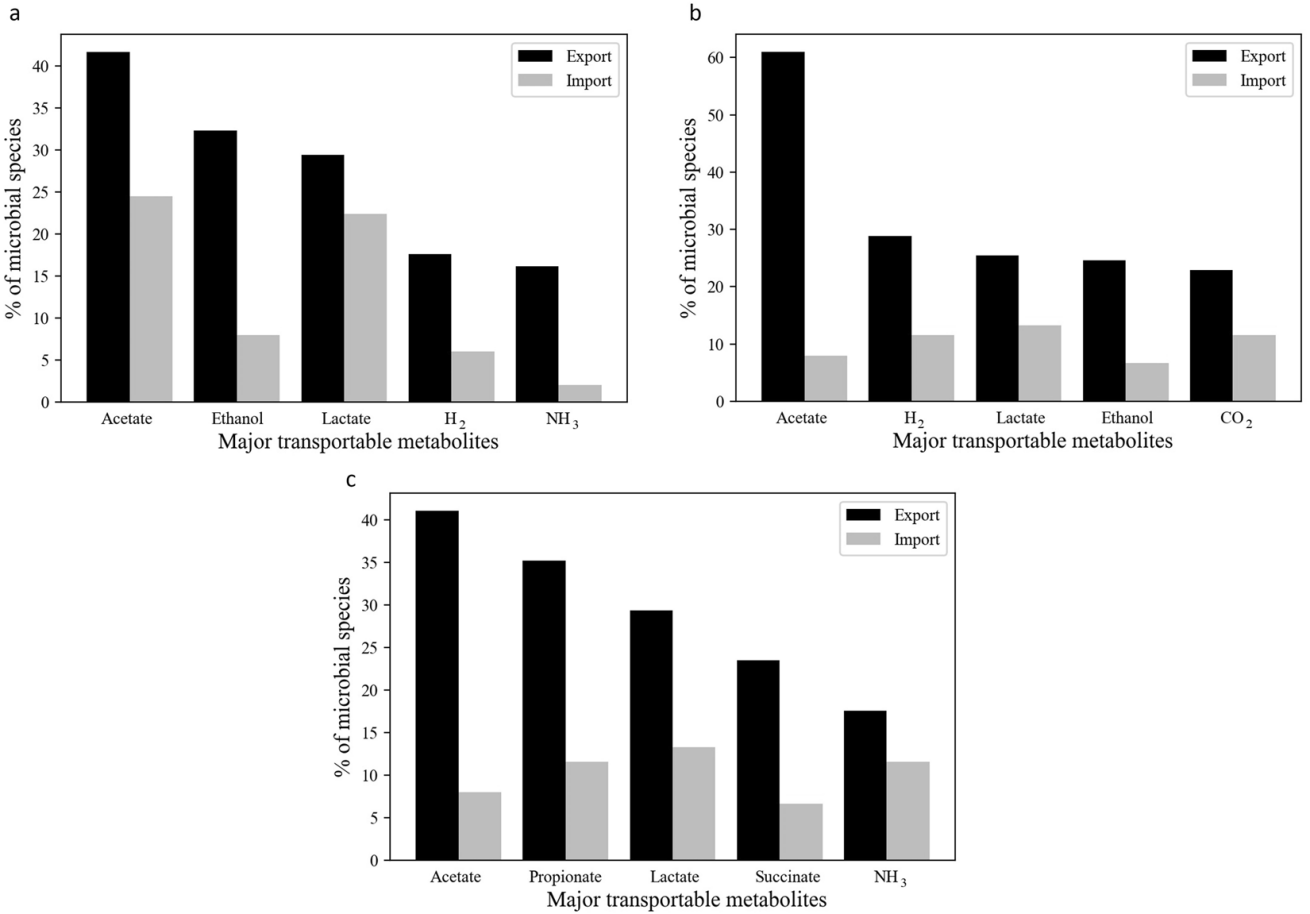
Frequently exported microbial byproducts and their consumption profile in different gut segments of *N*. *corniger*. The percentage of metabolic byproducts formation/consumption has been evaluated based on the number of microbial producers/consumers in different gut segments of *N*. *corniger*. (**a**) Five most commonly exported metabolic byproducts in segment S1 are acetate, ethanol, lactate, hydrogen, and ammonia. (**b**) In case of S2 segment five major exported metabolic byproducts are acetate, hydrogen, lactate, ethanol, and carbon dioxide. (**c**) Furthermore, acetate, propionate, lactate, succinate, and ammonia are five most predominant metabolic byproducts in S3 segment. Detail information of the metabolic consumptions and productions has been provided in Table S4.

The typical system of lignocellulose digestion and subsequent fermentation produce several crucial metabolic byproducts that drive the whole metabolic processes in the *N*. *corniger* gut. The distribution pattern of five most commonly produced metabolites in segment S1, S2, and S3 were quantified based on the number of microbial producers and consumers as represented in the inter-species influence network (Table S4). The frequency of metabolite production and consumption of microbes varied considerably in each gut segment. The acetate was produced by 41.7% of the microbial species in S1 segment, whereas the frequency of acetate producers was significantly increased to 61% in S2 segment. On the other hand, the frequency of acetate consuming microbes was decreased from 24.48% in S1 to 8% in S2. The result indicates a higher fermentation rate and the accumulation of acetate in S2 as compared to S1 (Fig. 5a). Apart from acetate, the formation of metabolic byproducts like ethanol, CO_2_, and H_2_ by numerous microbial species also indicates a significant amount of fermentation in S2 (Fig. 5b). However, the diversity of microbial species was less in the S3 segment (P5) and the commonly produced metabolites also deviated in comparison to S1 and S2 segments. In segment S3, 35.2% and 23.5% of microbes were involved in propionate and succinate production process, respectively, while the frequency of acetate producers reduced to 41.1% (Fig. 5c). The above results indicate that acetate is the most dominating metabolic byproduct throughout all the gut compartments of *N*. *corniger*, which is in agreement with previous physiochemical study of *N*. *corniger* gut environment^37^. However, acetate can also serve as a metabolic substrate for numerous species in the microbial community. Thus, in the absence of the conventional carbon sources, microbes can consume acetate to maintain the viability of the microbial niche and expedite the acetate removal from gut microenvironment. For example, some acetate consuming microbes like *Acinetobacter baumannii*, *Amycolatopsis pretoriensis*, *Celeribacter indicus*, *Fibrobacter intestinalis*, and *Heliobacterium modesticaldum* have been encountered in the microbial machinery of the *N*. *corniger*. Collectively, the study of metabolic byproduct distribution gave insight into the hierarchical metabolic function of dominating microbes inside the distinct gut compartment of *N*. *corniger*.

### Lignocellulose degradation network with an insight into enzyme machinery

**Figure 6:**
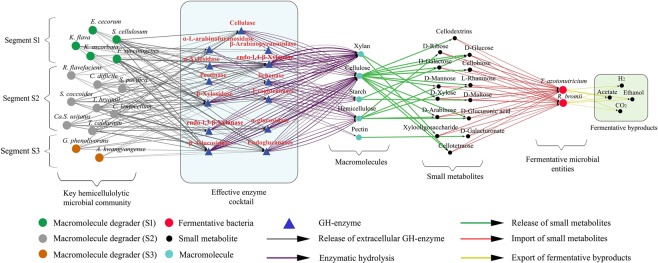
Lignocellulose degradation network. The network illustrates the process of macromolecule degradation and subsequent fermentation inside the *N*. *corniger* gut. The influential hemicellulolytic microbes (large green, orange, and gray-colored nodes) release extracellular enzymes (blue triangle) in their microenvironment denoted with the grey arrow. The effective enzyme cocktail act on macromolecule (deep cyan node) to produce small metabolites (small black nodes) as community feed. Fermentative microbial entities (large red nodes) can uptake small metabolites from the microenvironment to produce essential fermentative end products for maintaining the viability of a dynamic microbiota. Edges are weighted according to the confidence of a particular interaction.

Reconstruction and analysis of inter-species influence network helped us to identify 15 major lignocellulose degraders in *N*. *corniger* microbiota. The microbial processes of cellulose and hemicellulose degradation entirely depend on their enzymatic machinery in extracellular environment. The information of the extracellular GH enzymes produced by the key macromolecule degraders were collected from the CAZy database^38^. Hotpep and HMMER dbCAN2 Meta Server^39,40^ were used to annotate the CAZy domains for newer microbial genera. The sequential events of enzymatic hydrolysis and fermentation processes have been illustrated by the construction of lignocellulose degradation network with subsequent annotation of the hemicellulolytic enzyme cocktail (Fig. 6). The network exemplified the operational principle of a micro-community which acts collaboratively to accomplish complex metabolic events. The group of 15 lignocellulolytic microbes behaves like a micro-community that produces several active GH enzymes in their microenvironment. Glycoside hydrolases like endoglucanases, exoglucanases, endo-1, 4-β-xylanase, β-glucosidase, β-xylosidase, and cellobiohydrolase contributed as the major enzyme in the microbial enzymatic cocktail. This collaborative enzymatic machinery effectively breaks the complex plant polymers and provide simpler sugar for the community feed as described in the network. Fermentation of the simple sugar was also included as an extension of metabolic activity inside *N*. *corniger’s* microbiota. *R*. *bormii* and *T*. *azotonutricium* were found to be the dominant players in the fermentation process. These crucial fermentative microorganisms can uptake various small molecules such as dextrin, fructose, glucose, xylose, and ribose, to produce fermentative byproducts like H_2_, acetate, and CO_2_ in their microenvironment. Furthermore, the organic acids such as acetate, butyrate, and propionate, were produced during the process and absorbed in the *N*. *corniger* gut to support its sustainability^41,42^.

## Discussion

Wood-feeding higher termites have been considered as one of the most effective natural lignocellulose degraders. The lignocellulose degrading capability of termites has been achieved through the specialized microbial symbionts and their enzymatic machinery. To understand the integral system of natural lignocellulose digestion, we have developed a species-wide metabolic interaction network of *N*. *corniger’s* gut microbial communities. In order to assess the metabolic transport frequency, the metabolite import-export activity of each microbial entity have been thoroughly investigated. Determination of metabolic transportation frequency inside the gut microbiota indicates a higher tendency of metabolite consumption than production. The elevated level of metabolite consumption inside the gut microbial population triggered the competition among microbial groups having similar metabolic inputs. This metabolic competition plays a crucial role in maintaining the abundance of diverse microbial species and stabilize the microbial community composition^43,44^. This dynamic stability of the microbial community is possibly achieved through the evolution of the *N*.*corniger* and its microbiota. The metabolic competition was further investigated by calculating the correlation of metabolic similarity indices and microbial co-occurrence score. Interestingly, Spearman’s correlation coefficient indicates a positive correlation (rho = 0.305, *p* value 3.29 × 10^−12^) between the groups of lactic acid bacteria and propionic acid bacteria despite their similar metabolite uptake profile. The positive co-occurrence signifies that the gut physiological condition of *N*. *corniger* (host) is responsible for controlling the distribution pattern of the microbial population. The phenomenon of this host driven structure of the microbial community was previously characterized as habitat filtering^17,27,29^. In a metabolically competing microenvironment, microbes with a similar metabolic profile tend to control their co-occurrence negatively but habitat filtering act as a driving force to unite these competing microbial communities. For instance, the anaerobic condition in the hindgut punch of *N*. *corniger* promotes the growth of fermentative microbial communities in a significant manner^11,37^. Furthermore, this unique physiological condition of the hindgut punch is vital for establishing the dynamic stability of diverse microbial consortia. Apart from the metabolic competition, metabolic interdependency was also detected by investigating the correlation between metabolic similarity and co-occurrence (rho = 0.235, *p* value = 1.115 × 10^−7^) of ethanol producers and lignocellulose degraders. Fermentative bacteria (ethanol producers), incapable of utilizing cellulose and hemicellulose, receive their primary metabolites by the metabolic actions of lignocellulose degraders, which justifies the phenomenon of metabolic dependency. In the lignocellulose rich microenvironment of *N*. *corniger* gut, the metabolic dependency is accountable for a growth-promoting effects on microbial population, whereas metabolic competition acts as a substantial growth repressor. The dynamic effects of these contrasting metabolic phenomena was further investigated by inter-species influence network. In accordance with several earlier studies^15,31,45^, our study has also demonstrated the biological significance of microbial metabolic communication in a nutritionally interconnected microenvironment.

In a global arrangement of metabolic communication, the dynamic metabolic action of each species can affect the growth and viability of several microbes in either positive or negative manner. Inter-species influence network was constructed by evaluating the pairwise microbial interaction score (*I*_*pq*_) inside the gut microbiota. Betweenness centrality (*C*_*b*_) of 125 nodes were calculated to measure the overall influential role of each microbial entity over its community members. Key microbial players employing robust metabolic influences over a wide range of individuals were selected based on *C*_*b*_ value cutoff (>0.0074). The metabolic profiles of microbial species suggest that over 57% macromolecule degraders account for the crucial network influencers. Microorganisms like *C*. *lentocellum*, *F*. *succinogenes*, and *S*. *coccoides* exert effective positive metabolic influence by converting cellulose, hemicellulose, and starch into accessible community substrates like glucose, xylose, and cellobiose. In contrast, fermentative microbes (22%), *e*.*g*., *R*. *bromii* and *T*. *azotonutricium*, are observed to be an effective growth regulator, employing a strong negative metabolic influence by enhancing the metabolic competition through a wide range of substrate utilization. Both positive and negative influences are equally essential for the viability of a functional gut microbiota. The positive metabolic influence accounts for a substantial growth enhancement, whereas negative influence controls the growth rate of diverse microbial communities in order to balance the species abundance in a microbial niche. Hence, the combined effects of these positive and negative metabolic influences are responsible for maintaining a stable and functional gut microbiota, essential for the viability of *N*. *corniger*.

Furthermore, the contributions of different microbial communities involved in specialized metabolic processes were determined in distinct gut compartments of *N*. *corniger*. The frequency of acetogen was increased predominantly from 41% in segment S1 to 61% in segment S2. The most frequently produced metabolites in S2 segment are H_2_, lactate, ethanol and CO_2_ as a consequence of predominant fermentative bacterial genera like *Clostridium sp*., *Treponema sp*., and *Spirochaeta sp*. Distribution of microbial entities signifies a higher fermentation rate in segment S2 as compared to S1. Interestingly, a higher consumption rate of H_2_ and CO_2_ by dominating *Treponema sp*. indicates H_2_ dependent reductive acetogenesis process in S2 segement, which is crucial for the removal of metabolic byproducts. Apart from fermentation, macromolecule degradations were also found to be predominant in S2 segment, where 60% of the microbes have the abilities to degrade complex macromolecules. Hindgut punch (P3) of *N*. *corniger* has been reported as the most active gut compartment in terms of macromolecule degradation and fermentation^10,46^. Similarly, the S2 segment consisting of P3 and P4 compartments in the inter-species influence network exhibits the most diverse and enriched microbial population with a high frequency of macromolecule degraders and fermentative organisms. The anaerobic environment of the S2 segment is one of the main contributors towards the nourishment of highly active microbial machinery. However, the S3 segment does not actively take part in the fermentation processes^35^, while a moderate frequency of acetogenic microbes were observed along with some propionate and succinate producers. The overall mapping of the metabolic flow inside the gut compartments signifies the occurrence of several biochemical events in a synchronous manner. The macromolecule degradation and subsequent fermentation were found to increase from S1 segment, reached at maximum level in the most active S2 segment, and finally get declined in the terminal S3 segment. Hence, this inter-species metabolic communication leads to a chain of metabolic events for accomplishing the complex task of lignocellulose degradation with subsequent fermentation.

Although this metagenome based approach of microbial interactions study provides a system level understanding of the lignocellulose degradation inside termite gut, it has a few limitation. The metagenomics data of *N*. *corniger’*s gut segments were obtained from a single study^12^, which may introduce some biases in the microbial community composition. Diversity of the termite sample collection sites may provide some additional microbiological information, which is lacking in this study. Published experimental data of microbial metabolic activities provides the informational architecture of the network. However, experimental data were mostly generated in the straightforward *in vitro* laboratory condition and may often diverge from the complicated *in vivo* gut environment. Furthermore, each edge of our network represented the connection between metabolites and microbes based on the information of either the presence or absence of the corresponding metabolic associations. Therefore, the quantitative values of microbial transport reactions and the growth requirement cannot be recognized from our network. This species-wide metabolic interaction network should be contemplated as a global survey of metabolic phenomena inside a diverse microbial niche, instead of species-specific metabolic pathways. Although we acclaimed these drawbacks and limitation, we believe that these challenges will provide an essential guideline for the improvement of the network. Recognizable literature references and the confidence scores have been provided for each edge in the network, which will get improved and updated with further experimental evidence.

Detail comprehension of the metabolic activities among diverse microorganism inside natural bioreactor system of termite gut will be helpful to develop microbial community-based approaches for biofuel production^47,48^. *In silico* investigations of the microbial interaction pattern in this work can be considered as a progressive step towards this direction. Deliberate investigation of these inter-species metabolic influences may help in designing a stable microbial community by combining the co-operative and competitive phenomena. Improvisations of certain high-throughput quantitative techniques will help in *in vitro* and *in vivo* manipulation of the community metabolic influences to study the structural compatibility of a micro-community for achieving specialized functionality like lignocellulose degradation. Specifically, we have denoted the enzymatic cross-play of microbial communities, which is the basis of the bioreactor system inside *N*. *corniger’s* gut. Several crucial and predominant glycoside hydrolases with corresponding microbial producers in the termite gut will provide insights to develop co-culture-based lignocellulose bioreactor system. The enzyme cocktail obtained from lignocellulose degradation network may also assist in optimizing the enzymatic scarification step in the process of commercial biofuel production.

## Methods

### Metagenome sequence analysis of N. corniger gut microbiota to identify microbial species

Shotgun metagenome sequencing data of crop, midgut, and hindgut compartments of *N*. *corniger* were obtained from the IMG/M (JGI) metagenome repository portal (IMG Genome ID: crop (C) - 3300001542, midgut (M) - 3300001466, hindgut (P1) - 3300002238, hindgut (P3) - 3300002119, hindgut (P4)- 3300002308, and hindgut (P5) - 3300001343)^12^. Quality checked FASTQ sequence data were analyzed through Kaiju metagenome analysis server that uses the Burrows-Wheeler transform (BWT) algorithm to identify maximum exact matches at the proteome-level^49^. Kaiju had transformed the nucleotide sequence into amino acid sequence followed by fragmentation at the stop codon. The fragmented sequence was then sorted and searched against the reference protein database (103 million protein sequences) by backward search algorithm on the BWT^50,51^. Taxon identifiers of the corresponding database sequence were retrieved upon maximum exact matches with submitted metagenome sequence of *N*. *corniger* microbiota. The species-level information of the termite gut microbiota of different gut segments were retrieved with the MEM run mode. The abundance of microbial species in the distinct gut segment of *N*. *corniger* was determined using Krona^52^, an interactive metagenome visualization tool integrated with Kaiju. After analyzing the segment-specific sequence data, Kaiju produced multiple hits with diverse microbial population, wherein the 250 microbes were selected based on the considerable abundance values (≥0.02%) (Supplementary Data).

### Metabolic information collection and construction of species-wide interaction network

Out of the 250 identified microbial species, experimental information on metabolic activities were retrieved for 205 microbes and incorporated in the species-wide metabolic interaction network. About 400 published scientific research journals (Supplementary Data) were rigorously studied and evaluated to assemble the experimental data of all possible metabolic activities of identified microorganisms. Kyoto Encyclopedia of Genes and Genomes (KEGG) database^53^ have also been utilized to validate the collected metabolic information for certain microbes. Moreover, the confidence score of the metabolic information has been calculated to increase the reliability and transparency of our study (Supplementary Data). The primary or central metabolites, responsible for carrying out the intrinsic physiological properties like growth, development, and reproduction were considered as the small molecules in the network. Import and/or export of small molecules produced by macromolecule degradation were also precisely evaluated and annotated. A total of 10 macromolecules like cellulose, hemicellulose, pectin, and starch were included as a basic source of substrates inside *N*. *corniger* gut. A single macromolecule can be degraded by numerous species. Hence, the corresponding degradation products were considered as export metabolites of all the microbes participating in that particular macromolecule degradation. Secondary metabolites like quorum-sensing molecule and the toxic chemical compounds derived from primary metabolism were eliminated. Additionally, gaseous metabolites like H_2_, CO_2_, and hydrogen sulfide that can easily disperse through the cell membrane and influence the metabolism were also considered. In order to maintain the interaction network at the species level, all the annotated metabolic activities of different strains of a single species were merged as collective metabolic property.

#### Confidence score of species-wide interaction network

The experimental evidence, based on published literature, were the basic informational constituents of our network along with the KEGG database. However, while collecting the experimental evidence, we often observed biases in the available data for most common and well-studied microorganisms. We have assigned a confidence score for each data point in the network, which will increase the reliability of this study^54^. The confidence score of each edge was calculated based on the number of annotated literature along with the information available in the KEGG database. The confidence scores were determined by following equation:

1 $${\rm{Confidence}}\,{\rm{score}}=(\frac{n}{N}\ast 70)+(K\ast 30)$$

where N is the maximum number of literature annotations available for a single edge, n is the actual number of publication available for a particular edge and K denotes the availability of information in KEGG database. As we predominantly used the literature information to construct the metabolic interaction model, a high weightage (70%) was assigned to it, whereas a lower weightage (30%) was assigned to the KEGG database information. The value of the K can be either 1 or 0 based on the KEGG data availability or unavailability, respectively. The confidence scores of all the edges have been provided in the Supplementary Data (Sheet name: Confidence Score).

### Classification of gut segments based on microbial abundance distribution

The intestinal tract of *N*. *corniger* is compartmentalized into Crop (C), midgut (M), and hindgut (P1 to P5) segments^37^. Analysis of available metagenomic data of M, C, P1, P3, P4, and P5 segments provided information about diverse microbial communities. The abundance of microbial entities varied throughout different gut segments of *N*. *corniger* (Supplementary Data). In order to determine the level of abundance divergences among adjacent segments, the non-parametric Kruskal-Wallis one-way test (significance level 0.05) was carried out with compartment-specific sequence data^55^. Heat statistic and *p* value were calculated with a different combination of adjacent segments to justify the similarity of microbial abundance. The detail statistical formulation of the Kruskal-Wallis test is as follows:

2 $${\rm{H}}=(\frac{12}{{N}({N}+1)})\ast \sum \frac{{R}_{i}^{2}}{{n}_{i}}-3(N+1)$$

where *H* is the heat statistic, *R*_*i*_ is the rank of all observations in group *i*, *n*_*i*_ is the number of observations in group *i*, and *N* is the total number of observations. The microbial abundance in the crop, midgut, and P1 compartments suggested a positive correlation with each other, and they were considered as a single segment S1. Similarly, P3 and P4 compartment were taken together as the S2 segment while the P5 compartment was considered as a separate segment (S3). Hence, the gut segments of *N*. *corniger* were redefined as S1, S2, and S3 segments based on microbial abundance variation. Details of the statistical calculations have been provided in the Supplementary Information.

### Construction of inter-species influence network

Species-wide metabolic interaction network of microbial entities and metabolites were used as a fundamental prototype to construct an inter-species influence network. The mono-layered network of microbial metabolic influences illustrate the contribution of each microbial entity for maintaining a dynamic and functional gut microbiota. Communication between two interacting species leads to positive, negative, and neutral interaction giving rise to win, loss, and non-effective outcomes, respectively^56,57^. This elementary concept was acquired to construct the model of microbial influence on a global scale. In the gut microbial ecosystem, the cross-feeding activity of one microbe can stimulate the growth of beneficiary partner by supplying essential nutrients. This phenomenon was considered as a positive metabolic influence. On the other hand, two microbial entities can compete with each other to get better access to a common nutrient, thus limiting the available nutrient in their microenvironment. These competitive characteristics were responsible for negative or growth-inhibitory influence. Information from species-wide metabolic cross feeding network along with the combinational effects of positive and negative influences were utilized to quantify the net metabolic influence of a microbial entity *p* on another entity *q*. If we consider a pair of species *p* and *q*, an increase in *p*’s abundance may contribute to an increase or decrease in *q*’s abundance. We assume it to be *q*’s growth rate, or *q*’s abundance as they are directly related with each other. If each microbial entity *p* or *q* represents a single microbial species, the influence score can be calculated as^32^,

3 $${I}_{pq}=\frac{\partial {\mu }_{q}/{\mu }_{q}}{\partial {n}_{p}/{n}_{p}}=\frac{{n}_{p}}{{\mu }_{q}}\ast (\frac{\partial {\mu }_{q}}{\partial {n}_{p}})=\frac{{n}_{p}}{{\mu }_{q}}\sum _{m}\frac{\partial {\mu }_{q}}{\partial {x}_{mq}}\frac{\partial {x}_{mq}}{\partial {n}_{p}}$$

where *n*_*p*_ is the abundance of the microbial entity *p* and *µ*_*q*_ is the growth rate or abundance variation of entity *q*. Each metabolite consumed by species *q* is denoted by *x*, where *x*_*mq*_ is the consumption rate of metabolite *x* by unit abundance of *q* entity. Altogether, if the growth-promoting ability of microbial entity *p* is greater than its growth-inhibiting ability towards another microbial entity *q*, the net metabolic influence of *p* is considered as a positive influence, *i*.*e*. *I*_*pq*_ > 0. In contrast, if the growth-promoting ability of *p* is lesser than its growth-inhibiting ability, the entity *p* will have a net negative metabolic influence on the entity *q*, *i*.*e*. *I*_*pq*_ < 0.

The sampling analysis was also performed to determine the robustness of the inter-species influence network. In the first sampling analysis, we have reduced the number of both metabolites and microbial entities by 25%, compared to the original inter-species influence network. Whereas, in the second analysis, we have reduced only the microbial species information by 25%. The modified inter-species influence network I and II were regenerated with reduced metabolic information followed by network analysis (Figs S3 and S4). Furthermore, we have compared the modified and the original inter-species influence network, in order to assess the similarities between them (Tables S2 and S3). All the pairwise influence values have been recalculated for both of these modified influence network and provided in the Supplementary Data.

### Identification of influencer from inter-species influence network

Microbial influence network represents a comprehensive sketch of the inter-species influence properties based on their metabolic profile and abundance data. Topological network parameters like degree distribution and betweenness centrality were estimated to quantify the community scale metabolic influence of each microbial entity. The direct influence of a node *p* was quantified through the out-degree distribution parameter *i*.*e*., the total number of outwards edges. Hub nodes in the network were defined with a high number of outwards edges. Alternatively, the microbial entity *p* can also apply an indirect metabolic influence on any other entity *q* through in-between elements despite any direct connection. Potential network influencers were identified by calculating betweenness centrality of all nodes^58^. The betweenness centrality index (*C*_*b*_) of a node *p* is expressed as,

4 $${C}_{b}(p)=\frac{{\sum }_{x\ne p\ne y}({\sigma }_{xy(p)}/{\sigma }_{xy})}{(P-1)(P-2)}\ast 2$$

where *x* and *y* denote the non-identical nodes in the network different from *p*, σ_*xy*_ signifies the number of shortest paths from *x* to *y*, σ_*xy*_ (*p*) is the number of shortest paths from *x* to *y* through *p*, and *P* denotes the total number of nodes in the attached string that *p* belongs to.

### Construction of lignocellulose degradation network

Construction of species-wide metabolic interaction network and the analysis network topological properties helped us to identify about 47 different lignocellulose degraders in *N*. *corniger* gut microbiota. Further, the microbial influence (*I*_*pq*_) in the inter-species influence network revealed 15 most influential lignocellulose degraders like *Ca*. *S*. *usitatus*, *C*. *lentocellum*, *F*. *succinogenes*, *and S*. *coccoides*. CAZy database^38^ was rigorously searched to identify the effective glycoside hydrolases (GH) from these 15 crucial lignocellulose degraders involved in effortless degradation of cellulose and hemicellulose (Supplementary Data, Sheet name: lignocellulose degraders). Additionally, Hotpep (Frequency >2.6, Hits >6) and HMMER dbCAN2 Meta Server^39,40^ were utilized to annotate the CAZy domains for newer microbial genera. The information about microbial metabolic activities was sequentially combined and represented with distinct nodes and edges. The compilation of these diverse metabolic phenomena of specialized micro-communities eventually leads to the formation of lignocellulose degradation network. Extracellular hydrolysis of complex macromolecules, release of small metabolites, and the production of fermentative byproducts were sequentially illustrated in the lignocellulose degradation network.

## Supplementary information

Supplementary Information

Network Influencers, Enzyme database, Group wise abundance, Kruskal-Wallis test, Co-occurence matrix, Confidence_Score, Lignocellulose degraders, Metabolite import-export, References, Literature info

## Data Availability

Codes for computing the microbial co-occurrence and microbe–microbe metabolic influences are available on figshare (10.6084/m9.figshare.9770900.v1). The flow diagram of the overall methodology has been provided in the Supplementary information (Fig. S6). The authors declare that all other relevant data are available within the article and its Supplementary Information files, or from the corresponding author upon request.
