# Supplementary material for: Species-wide Metabolic Interaction Network for Understanding Natural Lignocellulose Digestion in Termite Gut Microbiota
Source: Sci Rep. 2019 Nov 8;9:16329. doi: 10.1038/s41598-019-52843-w (PMC6841923; doi:10.1038/s41598-019-52843-w)
Supplement: Supplementary file 1 — Supplementary Information [file 41598_2019_52843_MOESM1_ESM.pdf]

Supplementary Information

Species-wide Metabolic Interaction Network for Understanding Natural Lignocellulose Digestion in Termite Gut Microbiota

Pritam Kundu[1], Bharat Manna[1], Subham Majumder[1] and AmitGhosh[1,2*]

[1]*School of Energy Science and Engineering, Indian Institute of Technology Kharagpur, West Bengal, India-721302*

[2]*P.K. Sinha Centre for Bioenergy and Renewables, Indian Institute of Technology Kharagpur, West Bengal, India-721302*

*Corresponding author:
Dr. Amit Ghosh
Assistant Professor
School of Energy Science & Engineering
Sir J.C. Bose Laboratory Complex
Indian Institute of Technology Kharagpur
Kharagpur 721302,
WestBengal, India
Email: amitghosh@iitkgp.ac.in
Ph: +91-3222-260804

Analysis of metabolic activities from the structural properties of species-wide metabolic interaction Network

The probability of a random species for consuming 'm' metabolites has been calculated, which follows an exponential distribution: P (m) ∝ $e^{-r.m}$ (rate parameter r is 0.09 and 0.2 for import and export, respectively). A random metabolite is imported by 9 species and exported by 4 species (median 7 and 4 species, respectively). The probability that a given metabolite is imported and/or exported by 'z' species follows power-law distribution: P (z) ∝ m^{-y} (exponent y is 1.42 and 2.83 for import and export, respectively). Glucose is the most commonly used substrate in the microbiota. A total of 142 microbes utilize glucose either directly, or through macromolecule degradation. On the other hand, acetate is the most commonly generated metabolic end product, and it is produced by a total of 109 microbes. Although the microbial metabolite transportation is an uneven phenomenon, the above result indicates that the tendency of importing a metabolite is much higher than exporting by a given microbe within the microbial ecosystem.

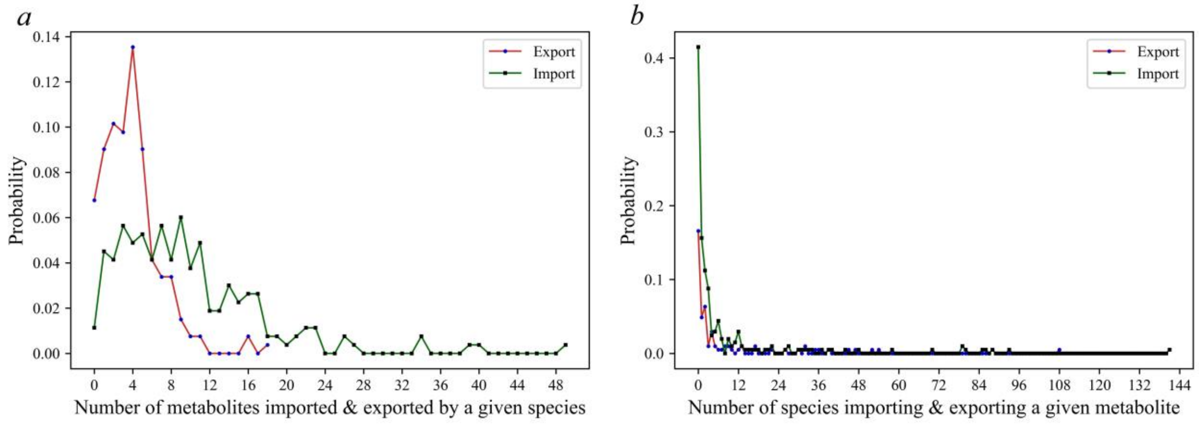

Figure S1. Analysis of network structural properties. (*a*) The plot demostrates the probability distribution 'P(m)' of a microbial species to import (red line) or export (green line) 'm' metabolites. The vertical and horizontal axis represent the probability distribution and the number of metabolites imported or exported, respectively. Both import and export follow the exponential distribution: P(m) ∝ $e^{-r.m}$, rate parameter r is 0.09 and 0.2 for import and export, respectively. (*b)* The plot of probability distribution 'P(z)' of a given metabolite, imported or exported by 'z' species. Here, the horizontal axis denotes the number of species that import (red line) or export (green line) a given metabolite while the vertical axis represents the probability, that follows the power-law distribution: P(z) ∝ m^{-y}, exponent y is 1.42 and 2.83 for import and export, respectively.

Correlation between the metagenomics abundance and the microbial metabolic import-export profile

We have calculated the correlation between the metagenomics abundance data and the combined import-export profile of the microbial species. The correlation coefficient (R^2 = 0.16) does not indicate any significant relation between metagenomics abundance and microbial metabolic

transport frequencies. The overall distribution of the microbial abundance is random inside the *N. corniger* gut.

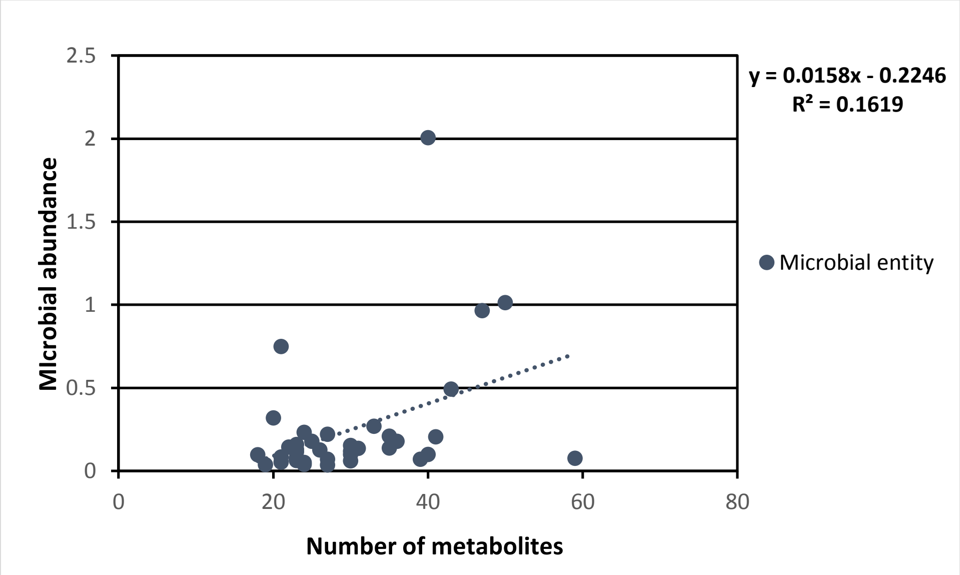

Figure S2. Correlation between microbial abundance and import-export profile: The vertical axis represents the distribution of microbial abundance, whereas the horizontal axis denotes the metabolite imported and exported by each microbe (black circle). The positive correlation value (R^2) was found to be 0.16 between microbial abundance and their metabolic transport profile.

Table S1. List of the promising microbial entities in terms of metabolic import and export profile and their total abundance

Microbial entity	Cosuming metabolite	Producing metabolite	Total Abundance
Acinetobacter baumannii	17	4	0.75
Aestuariimicrobium kwangyangense	19	4	0.115
Alistipes indistinctus	17	2	0.039
Alkaliflexus imshenetskii	18	6	0.053
Amycolatopsis pretoriensis	24	7	0.138
Arachidicoccus rhizosphaerae	23	7	0.12
Auraticoccus monumenti	22	5	0.072
Bacteroides fragilis	23	17	0.1
Bacteroides graminisolvens	17	10	0.036
Bacteroides ihuae	18	6	0.04
Bacteroides pyogenes	20	10	0.061
Burkholderia pseudomallei	17	8	0.179
Celeribacter indicus	41	6	0.967
Clostridium cellulosi	16	5	0.085
Clostridium difficile	27	12	0.072
Escherichia coli	24	19	0.493
Ethanoligenens harbinense	19	11	0.1
Fibrobacter succinogenes	18	17	0.14

Granulicoccus phenolivorans	50	9	0.077
Hamadaea tsunoensis	18	4	0.144
Kluyvera ascorbata	28	7	0.211
Lactococcus lactis	16	4	0.32
Mesorhizobium plurifarium	35	5	2.008
Micromonospora rosaria	21	5	0.128
Mycobacterium rhodesiae	20	4	0.234
Pelagibaca bermudensis	40	10	1.015
Pilibacter termitis	23	7	0.155
Prolixibacter bellariivorans	16	5	0.054
Propionibacterium cyclohexanicum	18	5	0.131
Pseudomonas aeruginosa	24	9	0.27
Pseudomonas fluorescens	27	9	0.18
Ralstonia solanacearum	35	6	0.206
Ruminococcus bromii	17	6	0.08
Sediminispirochaeta bajacaliforniensis	16	7	0.079
Sporomusa sphaeroides	17	6	0.16
Streptomyces nodosus	16	2	0.099
Tangfeifania diversioriginum	18	5	0.064
Treponema denticola	18	9	0.222
Youngiibacter fragilis	17	6	0.069

Statistical analysis for the correlation of metabolic similarity indices and the co-occurrence score

Relationship between the microbial abundance distribution and their metabolic profile have been calculated by adopting the statistical method used by *Roie Levy and Elhanan Borenstein*[2]. The metabolic similarity indices of each species-pair were calculated using the Jaccard similarity coefficient**.** For investigating the Jaccard similarity, sets of imported and exported metabolites have been calculated for two species *P* and *Q*. Where M_P is the set of metabolites for species *P*; M_Q is the set of metabolites for species *Q*; J_M is the metabolic similarity index between species *P* and *Q*. The Jaccard index has been calculated by the following equation:

$$J_M(M_P, M_Q) = \frac{|M_P \cap M_Q|}{|M_P \cup M_Q|} = \frac{|M_P \cap M_Q|}{|M_P| + |M_Q| - |M_P \cap M_Q|}$$

Similarly, the co-occurrence score for two species *P* and *Q* was obtained by calculating the Jaccard similarity coefficient of the microbial abundance. Where the A_P is the set of segment wise abundance for species P; A_Q is the set of segment wise abundance for species *Q*; J_A is the abundance similarity index (co-occurrence score) between *P* and *Q*.

$$J_A(A_P, A_Q) = \frac{|A_P \cap A_Q|}{|A_P \cup A_Q|} = \frac{|A_P \cap A_Q|}{|A_P| + |A_Q| - |A_P \cap A_Q|}$$

After evaluating the metabolic similarity index (J_M) and the abundance similarity index (J_A) for each species pair, the J_M and J_A values were stored in the form of metabolic similarity matrix and co-occurrence matrix respectively.

Now, each element of the co-occurrence matrix provides the co-occurrence score of a particular species pair, while elements of the metabolic similarity matrix provide the score of metabolic similarity indices. Thus, the components of the metabolic similarity matrix and the co-occurrence matrix were compared through the Spearman's rank correlation methods to investigate the relationship between the occurrence scores and metabolic similarly indices**.** Using this method, we have investigated the correlation between metabolic similarity and the co-occurrence score for several microbial groups like lignocellulose degraders, ethanol producers, lactic acid bacteria, and propionic acid bacteria. A positive correlation was observed (*rho = 0.235*) between the lignocellulose degraders and the ethanol producer with a significant *p* value of 1.115×10^{-7}, whereas the positive correlation score (*rho*) between the lactic acid bacteria and propionic acid bacteria was found to be 0.305 with a significant *p* value of 3.29×10^{-12}.

Kruskal–Wallis H test for compartment categorization for inter-species influence network

Categorization of Segment S1 (crop + midgut + P1): Microbial abundance data of crop, midgut, and P1 gut segments were organized in ascending order, followed by rank assignment to each of the values. In case of rank ties, the average value of the ranks were considered during the calculation (Supplementary Data: Sheet Name: Kruskal-Wallis test). The sum of ranks for crop, midgut, and P1 segments were found to be 99486 (R_1), 89873.5 (R_2), and 92265.5 (R_3) respectively.

The following null and alternative hypotheses were tested using the Kruskal-Wallis test[1],

H_o: The samples having populations with equal medians
H_a: The samples having populations with unequal medians

The rejection region for Chi-Square test, $R = \{X^2: X^2 > 5.991\}$, considering the significance level (α) as 0.05, and the degrees of freedom as 2.

The H statistic has been calculated using the following formula,

$$H = \frac{12}{N(N+1)} \times \left(\frac{R_1^2}{n_1} + \frac{R_2^2}{n_2} + \cdots + \frac{R_k^2}{n_k}\right) - 3(N+1)$$

$$= \frac{12}{750(750+1)}\left(\frac{99486^2}{250} + \frac{89873.5^2}{250} + \frac{92265.5^2}{250}\right) - 3(750+1)$$

$$= 4.268$$

Since it has been observed that $X^2 = 4.268 \leq X_U^2 = 5.991$ and the *p*-value 0.1183 ≥ 0.05 the null hypothesis H_o is not rejected. Therefore, there is sufficient evidence to claim that a significant fraction of the population medians were almost evenly distributed, at $\alpha = 0.05$ significance level.

Categorization of Segment S2 (P3 + P4):

The sum of ranks for segments P3 and P4 have been found to be 60433 (R_1) and 64817 (R_2) respectively. The rejection region for Chi-Square test, $R = \{X^2 : X^2 > 3.841\}$, considering the significance level (α) as 0.05, and the degrees of freedom as 1.

The H statistic has been calculated using the following formula,

$$H = \frac{12}{N(N+1)} \times \left(\frac{R_1^2}{n_1} + \frac{R_2^2}{n_2} + \cdots + \frac{R_k^2}{n_k}\right) - 3(N+1)$$

$$= \frac{12}{500(500+1)}\left(\frac{60433^2}{250} + \frac{64817^2}{250}\right) - 3(500+1)$$

$$= 1.748$$

Since has been observed that $X^2 = 1.748 \leq X_U^2 = 3.841$ and the *p*-value 0.1748 ≥ 0.05, the null hypothesis H_o is not rejected. Therefore, there is sufficient evidence to claim that a major fraction of the population medians were almost evenly distributed, at $\alpha = 0.05$ significance level.

Robustness of inter-species influence network:

The sampling analysis was performed by randomly reducing 25% of the original data. In the first sampling analysis, we have reduced the number of both metabolites and microbial entities by 25%. The original inter-species influence network (Figure 3) was regenerated by reducing 25% of metabolites and microbial entities, as shown in Figure S3. After analyzing the influence network, the results were compared with our original influence network. It was found that ~75% influencers retained their influencing characteristics in the modified influence network (Table S2). Assessment of the metabolic profile revealed that about 56% (57% in original influence network) of the influencers were exclusively involved in macromolecule degradation producing primary metabolites, whereas about 16% microbes (22% in original influence network) solely utilized sugars to produce fermentation products such as ethanol, butanol, H_2, and CO_2. More interestingly, 20% (11% in original influence network) of the influential microbes were capable of facilitating the task of both macromolecule degradation and fermentation. Furthermore, in our second analysis, we have reduced only the microbial species information by 25%. The original inter-species influence network (Figure 3) was regenerated, as shown in Figure S4. The modified network shows a 75% similarity with the original network in terms of network influencers (Table S3). Macromolecule degraders account for a significant fractions of network influencers in both modified influence network I and II which is similar to the original influence network. All the pairwise influence values have been recalculated for the modified inter-species influence network I and II (Supplementary Data, Sheet name: Modified Influence Network I).

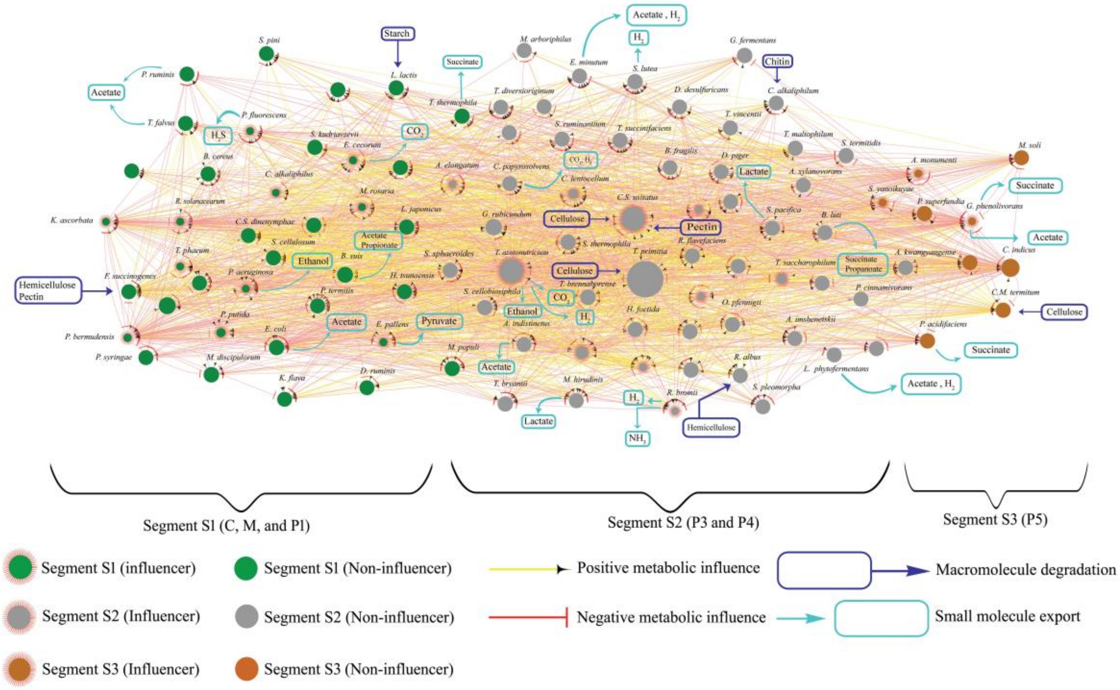

Figure S3. Modified interspecies influence network I with 25% reduction in metabolites and microbial information. The network represents an inter-species metabolic influence map of abundant microbial entities in S1 (green nodes), S2 (grey nodes), and S3 (orange nodes) gut segments of *N. corniger*. The modes of each pairwise interaction have been characterized as positive (yellow edges) or negative (red edges) metabolic influence. Nodes with extended red background signify as network influencers that employ considerable metabolic influence over a large number of individual entities. Moreover, diverse metabolic events of small molecule export (cyan box) and macromolecule degradations (blue box) are also annotated to describe the metabolic flow in the gut microbial machinery.

Table S2. List of the potential influencers in modified influence network with high betweenness centrality value (>0.0074). All the pairwise influence scores have been provided in the Supplementary Data (Sheet name: Modified Influence Network I).

Sl. no	Microbial species	Betweenness centrality
1	*A. elongatum*	0.0126
2	*A. monumenti*	0.0088
3	*Ca. S usitatus*	0.0144
4	*C. lentocellum*	0.0142
5	*C. alkaliphilus*	0.0085
6	*C. difficile*	0.0080
7	*D. cuneatus*	0.0207
8	*E. scecorum*	0.0184
9	*E. pallens*	0.0141
10	*G. phenolivorans*	0.0778
11	*K. ascorbata*	0.0291

12	*M. rosaria*	0.0170
13	*P. bermudensis*	0.0869
14	*P. aeruginosa*	0.0105
15	*P. fluorescens*	0.0076
16	*P. putida*	0.0096
17	*R. solanacearum*	0.0244
18	*R. bromii*	0.0196
19	*S. coccoides*	0.0148
20	*S. yanoikuyae*	0.0219
21	*T. phaeum*	0.0128
22	*T. azotonutricium*	0.0162
23	*T. caldarium*	0.0080
24	*T. saccharophilum*	0.0148
25	*C. indicus*	0.0528
26	*E. coli*	0.0441
27	*F. succinogenes*	0.0676
28	*R. flavefaciens*	0.0513

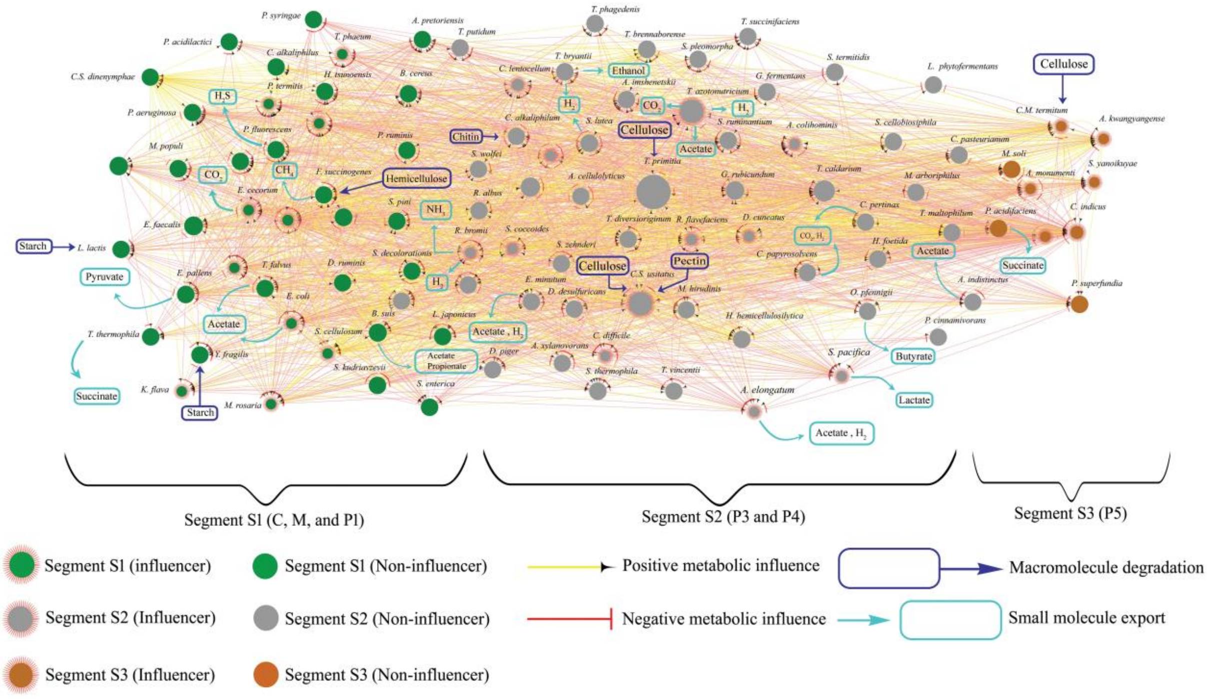

Figure S4. Modified interspecies influence network II with 25% reduction in microbial information

The network represents an inter-species metabolic influence map of abundant microbial entities in S1 (green nodes), S2 (grey nodes), and S3 (orange nodes) gut segments of *N. corniger*. The modes of each pairwise interaction have been characterized as positive (yellow edges) or negative (red edges) metabolic influence. Nodes with extended red background signify as network influencers that employ considerable metabolic influence over a large number of

individual entities. Moreover, diverse metabolic events of small molecule export (cyan box) and macromolecule degradations (blue box) are also annotated to describe the metabolic flow in the gut microbial machinery.

Table S3. List of the potential influencers in modified influence network with high betweenness centrality value (>0.0074). All the pairwise influence scores have been provided in the Supplementary Data (Sheet name: Modified Influence Network II).

Sl.no	Microbial species	Betweenness centrality
1	*A. elongatum*	0.0201
2	*A. kwangyangense*	0.0128
3	*A. colihominis*	0.0091
4	*A. monumenti*	0.0108
5	*B. fragilis*	0.0090
6	*Ca. M termitum*	0.0080
7	*Ca. S usitatus*	0.0147
8	*C. indicus*	0.0810
9	*C. lentocellum*	0.0199
10	*C. difficile*	0.0100
11	*D. cuneatus*	0.0180
12	*E. cecorum*	0.0281
13	*E. coli*	0.0296
14	*G. phenolivorans*	0.0599
15	*K. ascorbata*	0.0290
16	*K. flava*	0.0080
17	*M. rosaria*	0.0121
18	*P. bermudensis*	0.0931
19	*P. termitis*	0.0090
20	*R. cearum*	0.0161
21	*R. bromii*	0.0092
22	*R. flavefaciens*	0.0485
23	*S. pacifica*	0.0174
24	*S. cellulosum*	0.0177
25	*S. coccoides*	0.0075
26	*S. yanoikuyae*	0.0125
27	*T. phaeum*	0.0153
28	*T. azotonutricium*	0.0175

Table S4. The distribution of five most frequently produced metabolites and their consumption profile in S1, S2, and S3 segments.

Segment 1			Segment 2			Segment 3		
**Metabolites**	**Microbial producers (%)**	**Microbial consumers (%)**	**Metabolites**	**Microbial producers (%)**	**Microbial consumers (%)**	**Metabolites**	**Microbial producers (%)**	**Microbial consumers (%)**
Acetate	41.7	24.48	Acetate	61.01	8	Acetate	41.1	21.4

Ethanol	32.3	8	Hydrogen	28.81	11.6	Propionate	35.2	7
Lactate	29.4	22.4	Lactate	25.42	13.3	Lactate	29.4	21.4
Hydrogen	17.6	6	Ethanol	24.57	6.66	Succinate	23.5	7
Ammonia	16.2	2	Carbon dioxide	22.88	11.6	Ammonia	17.6	28.5

Table S5. A statistical overview of the network that includes total interactions, microbial species, abundance, metabolic uptake, and secreted metabolites.

**Overall statistical properties of the Species-wide metabolic interaction**	
Number of total microbial entity	205
Number of total metabolic compounds	265
Number of metabolic exchange events	2988
Number of consumption (import)	1776
Number of production (export)	565
Number of consumption (import) & production (export)	378
Number of macromolecule degradation	269
Average metabolic consumption	10.5
Average metabolic production	4.6

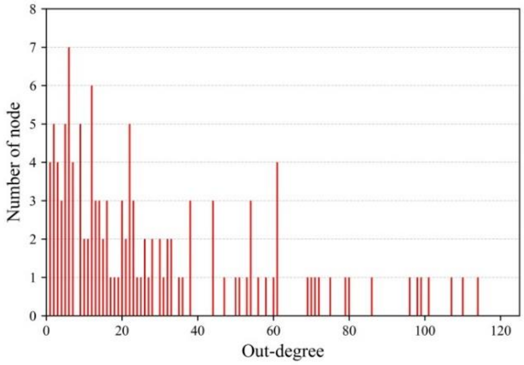

Figure S5. Frequency of out-degree distribution: Plot of out-degree distribution frequency of microbial entities for inter-species influence network. The horizontal axis denotes the number of outwards edges (out-degree) and the vertical axis signifies the total number of nodes possessing the same number of outwards edges. The number of nodes gradually decreases with an increase in out-degree values. Notably, very few nodes have a high out-degree parameter. For instance, *Treponema primitia* and *Celeribacter indicus* have an out-degree distribution of 110 and 114, respectively which indicates a greater direct influence on the community

Figure S6. Flow diagram of the overall methodology

Collection of metagenomics sequence data
IMG/M (JGI) metagenome repository portal: https://img.jgi.doe.gov/
Metagomic sequences used in this current study: IMG Genome ID-3300001542, 3300001466, 3300002238, 3300002119, 3300002308, 3300001343

↓

Analysis of Metagenomic data
The metagenomic sequences are analysed with Kaiju, a fast and sensitive taxonomic classification for metagenomics.
Server: http://kaiju.binf.ku.dk/
Input: Quality checked FASTQ sequence data
Output: Information of microbial entities (up-to species level)

↓

Metabolic information collection
400 published scientific research journals have been evaluated to assemble the experimental data microbial metabolic activities.
Kyoto Encyclopedia of Genes and Genomes (KEGG) database: https://www.genome.jp/kegg/

↓

Construction of species-wide metabolic interaction network
205 microbial species, 265 metabolites and 2988 metabolic events have been mapped using Cytoscape v3.6.1: https://cytoscape.org/
Network robustness calculation, Confidence Score calculation and Identification of community-level metabolic activity like cross-feeding interactions and metabolic competition.
Note: The detail network information has been provided in the ‘cytoscape’ directory, File name: ‘Species-wide_metabolic_interaction network_literature_infornation_as edge_attribute’

↓

Reconstruction of inter-species influence network
Inter-species influence network displayed the highly influential interactions of 125 microbes inside different gut segment of *N. corniger*. The quantitative value of each pair-wise metabolic influence has been represented in the ‘Ipq_Matrix’ (Supplementary data). ‘Ipq_matrix_calculation’ directory should be followed for accessing the code of Ipq_matrix generation.
Note: The detail network information has been provided in the ‘cytoscape’ directory, File name: ‘Inter-species_influence_network_metabolic_activity_as_edge_attribute’

↓

Identification of key influencers

Topological network parameters have been estimated to quantify the community scale metabolic influence of each microbial entity.

Parameter: Out-degree distribution and Betweenness centrality

Among all the microbial species represented in the influence network, 29.6% were found to be the most influential

↓

Assessment of Metabolic activity

57% of the influencers were exclusively involved in macromolecule degradation, 22% microbes solely utilized sugars to produce fermentation products and 11% of the influential microbes were capable of facilitating both tasks of macromolecule degradation and fermentation

↓

Lignocellulose degradation network with an insight into enzyme machinery

15 major lignocellulose degraders have been identified from *N. corniger* gut microbiota. The information of the extracellular GH enzymes has been collected from the CAZy database: http://www.cazy.org/

The CAZy domains for newer microbial genera have been annotated using Hotpep and HMMER dbCAN2 Meta Server: http://bcb.unl.edu/dbCAN2/blast.php

References:

1. Kruskal, W. H. & Wallis, W. A. Use of Ranks in One-Criterion Variance Analysis. *J. Am. Stat. Assoc.* **47,** 583–621 (1952).
2. Levy, R. & Borenstein, E. Metabolic modeling of species interaction in the human microbiome elucidates community-level assembly rules. *Proc. Natl. Acad. Sci.* **110,** 12804–12809 (2013).
